# Recent Advances in General Medicine

**Published:** 1897-06

**Authors:** 


### RECENT ADVANCES IN GENERAL MEDICINE.

Drs. William Pepper and Alfred Stengel, in reviewing the year's work upon this subject (*Amer. Year-Book of Med. and Surg.*, 1897), says that the present is undoubtedly an age of experimentation, and those who pursue this method of study reach the most useful results. Already the fruits of laboratory work are manifest in the greater accuracy of the knowledge of certain affections and in that which is more important to mankind, the discovery of principles that offer a justifiable hope of greater success in prevention and treatment of disease. In speaking of diphtheria the writers say that the same principles that have given to the world a remedy that may at least be assumed of probable value in this disease have been applied to other conditions. The work of Marmorek receives at their hands particular notice. Whether the serum prepared by this investigator will prove itself as useful in the treatment of erysipelas and other forms of streptococcus infection as some maintain or not, time alone can tell. At present the evidence favors a hopeful acceptance of the claims. In smallpox, typhoid fever, tuberculosis, and other infections, the results have been far less satisfactory, though flattering reports are not wanting. The authors refer to the greater accuracy of knowledge regarding various diseases. This is evidenced in the advance of bacteriology and the titles of reports of cases. Pyocyaneous infection, streptococcemia infection with the bacillus coli, etc., have taken the place of the more vague and insignificant titles—septicemia, pyemia, etc. Further investigations will doubtless establish more definite distinctions in the symptomatology and nature of the various infections; but for the present it is sufficient that they are beginning to be recognized and separately studied. Certain authors have maintained that acute rheumatism is a form of infection (micrococci), and the evidence is strong and suggestive. Reference may also be made to the positive demonstration of the gonococcus as a cause of general septicemia and of endocardiac and other metastatic lesions. Chemical investigators have been but little less active than the bacteriologists. The problems of auto-intoxication and the processes of normal and disordered metabolism are beginning to be understood, and special manifestations are recognized in which specific disturbances of the organic chemistry play an important part. There is the difference between the view of the present day and those of the older authors, that demonstration has taken the place of speculation. Reference need only be made in this connection to the investigations regarding myxedema and the thyroid gland, to the studies of uric acid formation, and to the investigations regarding the pathology of gout. Among other evidences of progress, attention may be called to the discovery of the specific cause of the bubonic plague by Kitasato, and the method of Elsner for the diagnosis of typhoid fever.—*A. and M. Bulleton.*

